# Centrosomal localization of RhoGDIβ and its relevance to mitotic processes in cancer cells

**DOI:** 10.3892/ijo.2012.1730

**Published:** 2012-12-07

**Authors:** YONG-SHENG JIANG, MASAYO MAEDA, MAYUMI OKAMOTO, MIKIKO FUJII, RYUICHIRO FUKUTOMI, MASATO HORI, MASAAKI TATSUKA, TAKAHIDE OTA

**Affiliations:** 1 Division of Tumor Biology, Department of Life Science, Medical Research Institute, Kanazawa Medical University; Uchinada, Ishikawa 920-0293;; 2 Department of Chemistry, Kanazawa Medical University, Uchinada, Ishikawa 920-0293;; 3 Department of Life Sciences, Life and Environmental Sciences, Prefectural University of Hiroshima, Shoubara, Hiroshima 727-0023, Japan;; 4 Oncology Department, Tongji Hospital, Tongji Medical College, Huazhong University of Science and Technology, Wuhan 430030, P.R. China

**Keywords:** Rho GDP-dissociation inhibitor, centrosome, cytokinesis, cancer progression

## Abstract

Rho GDP-dissociation inhibitors (RhoGDIs) are regulators of Rho family GTPases. RhoGDIβ has been implicated in cancer progression, but its precise role remains unclear. We determined the subcellular localization of RhoGDIβ and examined the effects of its overexpression and RNAi knockdown in cancer cells. Immunofluorescence staining showed that RhoGDIβ localized to centrosomes in human cancer cells. In HeLa cells, exogenous GFP-tagged RhoGDIβ localized to centrosomes and its overexpression caused prolonged mitosis and aberrant cytokinesis in which the cell shape was distorted. RNAi knockdown of RhoGDIβ led to increased incidence of monopolar spindle mitosis resulting in polyploid cells. These results suggest that RhoGDIβ has mitotic functions, including regulation of cytokinesis and bipolar spindle formation. The dysregulated expression of RhoGDIβ may contribute to cancer progression by disrupting these processes.

## Introduction

Rho family proteins function as molecular switches in various cellular processes, including actin cytoskeletal organization, microtubule dynamics, vesicle trafficking, cell cycle progression and cell polarity (1). More than 22 Rho family proteins have been identified in humans (2). There are three classes of regulators of Rho proteins, namely, Rho guanine nucleotide exchange factors (RhoGEFs), Rho GTPase-activating proteins (RhoGAPs) and RhoGDIs. In humans, over 69 RhoGEFs and 59 RhoGAPs have been characterized (3,4), while only three types of RhoGDIs (RhoGDIα/RhoGDI1, RhoGDIβ/RhoGDI2/LyGDI/D4GDI, and RhoGDIγ/RhoGDI3) have been identified (5). The existence of a great many types of RhoGEFs and RhoGAPs enables assignment of individual regulators to specific cellular processes. On the other hand, because there are fewer RhoGDIs, each type must regulate a wide range of cellular processes. Thus, RhoGDIs are considered multifunctional central regulatory molecules for Rho family proteins (5–8). This multifunctional nature of RhoGDIs makes it difficult to clarify their specific roles in various cellular events. RhoGDIα is a major RhoGDI and is universally expressed. RhoGDIγ is expressed in brain, lung and pancreas (9,10). RhoGDIβ was originally isolated as a RhoGDI that is abundantly expressed in hematopoietic cells (11,12), however, it is also expressed in several other cell types, including keratinocytes, fibroblasts, amnion cells (13), non-hematopoietic tumors (14–16) and in various normal human tissues (17). Therefore, RhoGDIβ is expected to have a more general cellular role, not specific to the hematopoietic cell lineage.

RhoGDIβ is implicated in cancer progression, however, reports have presented contradictory evidence as to the nature of the correlation between cancer progression and RhoGDIβ expression level. RhoGDIβ was found to be upregulated in ovarian cancer (18), breast cancer (19), gastric cancer (20), and in pancreatic cancer cells that show high perineural invasion (21,22). The full-length RhoGDIβ promotes cancer cell invasion (19) and survival (23) in human breast cancer. In our previous studies, RhoGDIβ lacking C-terminal region was identified to induce metastasis by activating the Rac1 signaling pathway in c-Ha-Ras-transformed fibroblasts (15,24). In other studies, RhoGDIβ has been reported to suppress invasion (14) and its expression is inversely correlated with invasive capacity in human bladder cancer cells (16). Our experiments showed that RhoGDIβ lacking the N-terminal regulatory domain suppresses metastasis by promoting anoikis in v-Src-transformed fibroblasts (25). Metastasis suppression by RhoGDIβ is enhanced by Src-induced RhoGDIβ phosphorylation (26) and correlates with increased Rac1 activity (27). Thus, indicating yet-undetermined roles in cancer cells, there are inconsistent results regarding RhoGDIβ expression and cancer progression.

To clarify the role of RhoGDIβ in cancer progression, in the present study, we examined the subcellular localization of RhoGDIβ and the effects of overexpression and RNAi knockdown of RhoGDIβ in cancer cells. We found that RhoGDIβ localized to centrosomes in human cancer cells. In HeLa cells, exogenous GFP-tagged RhoGDIβ localized to centrosomes and its expression resulted in prolonged mitosis and aberrant cytokinesis. Knockdown of RhoGDIβ increased the incidence of monopolar spindle mitosis and polyploid cells in HeLa cells. The resulting polyploid cells were possibly caused by perturbation of centrosomal function with a lack of RhoGDIβ. Our presented results give new insights about the role of RhoGDIβ in cancer progression.

## Materials and methods

### Cells and cell culture

The human cervical cancer cell line HeLa was provided by the late Professor Masakatsu Horikawa, Faculty of Pharmaceutical Sciences, Kanazawa University (Kanazawa, Japan) (28). The human colon cancer cell lines HT-29, HCT116 and SW48 were purchased from ATCC (Rockville, MD). The human colon cancer cell lines DLD-1 and LoVo were purchased from the Human Science Research Resources Bank (Osaka, Japan). The human colon cancer cell lines SW480 and SW620 (29) were provided by Dr Ryuichi Yatani, Mie University School of Medicine (Mie, Japan). These cells were cultured in Dulbecco’s modified Eagle’s medium (DMEM) containing 10% fetal bovine serum (FBS), penicillin (50 U/ml), and streptomycin (50 *μ*g/ml) at 37°C in a humidified atmosphere of 5% CO_2_ and 95% air. Human microvascular endothelial cells (HMVEC) (Kurabo Industries Ltd., Osaka, Japan) were maintained in HuMedia-MvG in accordance with the supplier’s instructions. Immortal OKF keratinocytes (OKF6/TERT-2) were kindly provided by Dr J.G. Rheinwald (Harvard Medical School, Boston, MA) and were cultured in keratinocyte serum-free medium (K-sfm, BD Biosciences, San Diego, CA) with 30 *μ*g/ml bovine pituitary extract, 0.1 ng/ml EGF and 0.4 mM Ca^2+^(30). This culture condition permits cells to form cadherin- and desmosome-mediated junctions, and for some cells to stratify.

### Antibodies

Anti-RhoGDIα antibody (sc-360), anti-RhoGDIβ antibody (sc-6047) and the blocking peptide (sc-6047P) for the anti-RhoGDIβ antibody (sc-6047) were purchased from Santa Cruz Biotechnology Inc. (Santa Cruz, CA). Anti-GFP (ab290), anti-γ-tubulin (clone GTU-88) and anti-RhoGDIβ (556498) antibodies were purchased from Abcam (Cambridge, UK), Sigma-Aldrich (St. Louis, MO) and BD Biosciences (San Jose, CA), respectively. The antigen peptides for the anti-RhoGDIβ (sc-6047) and anti-RhoGDIβ (556498) antibodies were amino acid residues 175–194 and 4–21, respectively, of human RhoGDIβ. Anti-RhoGDIα/RhoGDIβ antibody (66586E) was purchased from Pharmingen (San Diego, CA). Peroxidase-conjugated anti-mouse and anti-rabbit IgG antibodies were purchased from DakoCytomation (Glostrup, Denmark). Peroxidase-conjugated anti-goat IgG antibody was purchased from Nichirei Corporation (Tokyo, Japan).

### Preparation of mouse organs for immunoblotting

Six-week-old female ICR mice were obtained from Japan SLC Inc. (Shizuoka, Japan) and maintained under pathogen-free conditions. Seven-week-old mice were anesthetized with pento-barbital, then blood was gently drained from the inferior vena cava using a heparinized syringe equipped with a 22-gauge needle. Leukocytes and erythrocytes were isolated from blood using Lympholyte^®^-Mammal (Cedarlane Laboratories Ltd., Burlington, Canada) according to the manufacturer’s instructions and were lysed in Laemmli buffer (31). After blood collection, organs were quickly removed, washed with ice-cold phosphate-buffered saline (PBS), minced into small pieces and lysed in Laemmli buffer. All experiments using mice were approved by the Committee on Experimental Animals at Kanazawa Medical University and conducted in accordance with their guidelines.

### Immunoblotting

Protein concentrations of lysed cells and organs were measured using the Bradford ULTRA kit (Novexin Ltd., Cambridge, UK). Proteins were resolved by sodium dodecyl sulfate-polyacrylamide gel electrophoresis and transferred to Immobilon-P membranes (Millipore, Billerica, MA). The membranes were then probed with a primary antibody, followed by incubation with a peroxidase-conjugated secondary antibody. Immunoreactive proteins were visualized using ECL Plus reagents (GE Healthcare UK Ltd., Little Chalfont, UK). In the immunoblot experiments, we used RhoGDIα as a loading control because of its universal expression. We applied the same amount of protein for immunoblot experiments. The samples from organs contain several kinds of extracellular matrices at various levels and the cell numbers contained in each samples was not constant, therefore the expression levels of RhoGDIα among various organs were not as constant as those among cultured cell lines.

### Immunofluorescence staining

For immunofluorescence staining, cells were grown on 35-mm culture dishes (BD Biosciences, San Jose, CA) or Lab-Tek II chamber slides (Nalge Nunc International, Naperville, IL). Cells were fixed with freshly prepared 4% paraformaldehyde for 2 min, permeabilized with 0.5% Triton X-100 for 10 min and fixed again with 4% paraformaldehyde for 10 min at room temperature. In some experiments, cells were simply fixed with 99.8% methanol for 30 min. After washing with PBS, cells were incubated with 0.5% bovine serum albumin (BSA) in PBS for 60 min at room temperature and then incubated overnight at 4°C with primary antibodies diluted in 0.5% BSA in PBS (1:2,000 for anti-γ-tubulin antibody or 1:200 for all other primary antibodies). After three washes with PBS, cells were incubated for 60 min at room temperature with secondary antibodies (Invitrogen, Carlsbad, CA) diluted 1:400 with 0.5% BSA in PBS containing 0.1 *μ*g/ml 4′,6-diamidino-2-phenylindole (DAPI). After washing with PBS, cells were mounted with Prolong Gold (Invitrogen). For the blocking experiment, anti-RhoGDIβ antibody (sc-6047) was incubated with 10-fold concentration of the blocking peptide for 60 min at room temperature before use. Images were obtained using an Axiovert 200 inverted fluorescence microscope or LSM710 confocal microscope (Carl Zeiss, Jena, Germany).

### Plasmids and transfection

The entire sequence was amplified by PCR using pcDNA3.1-LyGDI, an expression vector for wild-type human RhoGDIβ (15). The product was then subcloned into pAcGFP1-C3 and used as pAcGFP-RhoGDIβ. Cells were transfected with the expression plasmids using Lipofectamine 2000 (Invitrogen). To obtain cell lines that stably expressed the introduced genes, G418-resistant cells were isolated in medium containing 800 *μ*g/ml G418 (Nacalai Tesque Inc., Kyoto, Japan).

### Observation of GFP-RhoGDIβ in living cells and time-lapse analysis

HeLa cells that stably expressed GFP-RhoGDIβ were cultured in 35-mm glass-bottomed dishes (Asahi Glass Co. Ltd., Tokyo, Japan). The cells were maintained at 37°C in a humidified atmosphere of 5% CO_2_ and 95% air in an enclosed stage incubator (Incubator-XL, Carl Zeiss) built on top of an Axiovert 200 M inverted microscope. Time-lapse images of green fluorescence and differential interference contrast (DIC) were acquired on an Axiovert 200 M controlled by AxioVision image processing and analysis system 4.4 (Carl Zeiss). The onset of mitosis was considered to be the beginning of cell rounding, the onset of anaphase was defined as the beginning of chromosome segregation and the end of cytokinesis was recognized when the cleavage furrow maximally contracted. We analyzed the progression of mitosis temporally in cells in which these mitotic processes could be clearly recognized. The entire duration of mitosis was measured as the time from the beginning of cell rounding to the maximal contraction of the cleavage furrow.

### Small interfering RNA (siRNA)

Three different 25-mer Stealth RNAi duplexes targeting human RhoGDIβ and negative control Stealth RNAi were purchased from Invitrogen. The sequences of the three Stealth RNAi are as follows: no. 153, sense 5′-GAGCUGGACAGCAAGCUCAAUUAUA-3′, anti-sense 5′-UAUAAUUGAGCUUGCUGUCCAGCUC-3′; no. 469, sense 5′-GCCUGAAAUACGUUCAGCACACCUA-3′, anti-sense 5′-UAGGUGUGCUGAACGUAUUUCAGGC-3′; and no. 800, sense 5′-GGUCCCUCUUCAACACUGCCACAUU-3′, anti-sense 5′-AAUGUGGCAGUGUUGAAGAGGGACC-3′. Stealth RNAi duplexes were transfected into HeLa cells using Lipofectamine 2000 according to the manufacturer’s protocol.

### DNA histograms

Cells were fixed with 20% ethanol and incubated with 0.1% RNase (Type II-A, Sigma-Aldrich, St. Louis, MO) for 30 min at 37°C. The cells were stained with propidium iodide (50 *μ*g/ml) and analyzed using a FACSort flow cytometer (BD Biosciences, San Jose, CA).

### Statistical analysis

Differences between values were analyzed by the two-tailed Mann-Whitney U-test using the statistics function of KaleidaGraph (Version 4.1). P<0.05 was considered significant.

## Results

### Protein expression levels of RhoGDIβ

**Figure 1.**
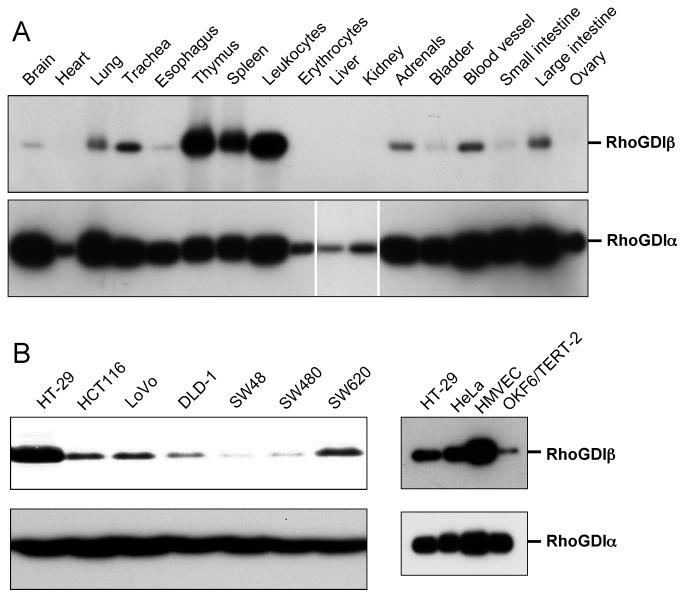
Protein expression levels of RhoGDIβ in mouse organs and cell lines. (A) Organs were collected from normal female mice and the levels of RhoGDIβ proteins were determined by immunoblot analysis using anti-RhoGDIβ antibody (sc-6047). Images of representative immunoblots from two independent experiments are shown. (B) Whole-cell lysates were prepared from the indicated human cell lines and the levels of RhoGDIβ protein were determined by immunoblot analysis using anti-RhoGDIβ antibody (556498). Images of representative immunoblots from at least three independent experiments are shown. RhoGDIα, which is a universally expressed RhoGDI, was stained using anti-RhoGDIα antibody (sc-360) as a loading control.

To confirm the previous observations showing the expression of RhoGDIβ in cells other than those in the hematopoietic cell lineage (13–17), we examined the levels of RhoGDIβ protein in various mouse organs (Fig. 1A, upper panel). RhoGDIβ was expressed in mouse brain, lung, trachea, esophagus, adrenals, bladder, blood vessels, small and large intestine, although the expression levels were much lower than in hematopoietic cells such as those from the thymus, spleen and leukocytes. Blood was thoroughly drained before the isolation of organs, but slight contamination of hematopoietic cells was unavoidable. Therefore, it could not be ruled out that very low expression of RhoGDIβ reflects contamination of hematopoietic cells. In contrast, RhoGDIα was expressed at various levels in all examined tissues (Fig. 1A, lower panel). Consistent with the expression of RhoGDIβ in the large intestine and in blood vessels, RhoGDIβ was expressed in cultured human colon cancer cells and microvascular endothelial cells (HMVEC) (Fig. 1B). RhoGDIβ was also expressed in other types of cultured epithelial cells, such as HeLa and OKF6/TERT-2 human keratinocytes (Fig. 1B).

### Subcellular localization of RhoGDIβ

**Figure 2.**
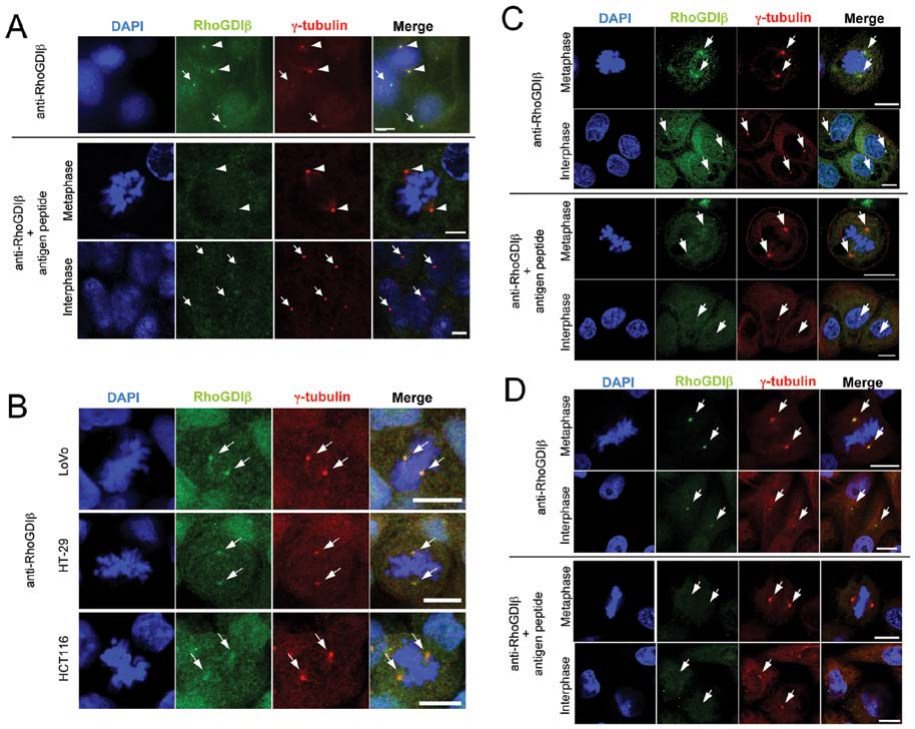
Colocalization of RhoGDIβ and γ-tubulin in cultured cells. (A) DLD-1 cells were fixed with 4% paraformaldehyde, permeabilized with 0.5% Triton X-100, fixed again with 4% paraformaldehyde, then stained simultaneously with anti-RhoGDIβ (sc-6047) (green) and anti-γ-tubulin (red) antibodies and with DAPI (blue) (upper panels). Pretreatment with antigen peptide abolished the staining by anti-RhoGDIβ antibody (lower panels). Arrows indicate centrosomes in interphase cells and arrowheads indicate centrosomes in mitotic cells. (B) LoVo, HT-29 and HCT116 human colon cancer cells were fixed and stained as in (A). Arrows indicate centrosomes. (C) OKF6/TERT-2 cells were fixed with methanol and stained as in (A) (upper panels). Pretreatment with antigen peptide abolished the staining by anti-RhoGDIβ antibody (lower panels). Arrows indicate centrosomes. (D) HeLa cells were fixed and stained as in (A) (upper panels). Pretreatment with antigen peptide abolished the staining by anti-RhoGDIβ antibody (lower panels). Arrows indicate centrosomes. Scale bars indicate 10 *μ*m. Representative images from more than three independent experiments are shown.

We examined the subcellular localization of RhoGDIβ in cultured colon cancer cells by immunofluorescence staining. Simultaneous staining with anti-RhoGDIβ and anti-γ-tubulin antibodies showed the colocalization of them in DLD-1 human colon cancer cells (Fig. 2A, upper panels). Similar results were also obtained in HT-29, HCT116, LoVo, SW48, SW480 and SW620 human colon cancer cell lines. Representative images of simultaneous staining with anti-RhoGDIβ and anti-γ-tubulin antibodies in LoVo, HT-29 and HCT116 human colon cancer cells are shown in Fig. 2B. RhoGDIβ colocalized with γ-tubulin also in OKF6/TERT-2 human keratinocyte (Fig. 2C, upper panels) and HeLa cells (Fig. 2D, upper panels). During interphase, the colocalization of RhoGDIβ with γ-tubulin was not as clear as during metaphase. Such centrosome staining pattern is likely to be associated with centrosome maturation. The staining with anti-RhoGDIβ antibody was abolished by pretreatment of the antibody with antigen peptide (Fig. 2A, C and D, lower panels). Unlike RhoGDIβ, RhoGDIα did not colocalize with γ-tubulin in colon cancer cells and HeLa cells (data not shown).

### Subcellular localization of GFP-RhoGDIβ in HeLa cells

**Figure 3.**
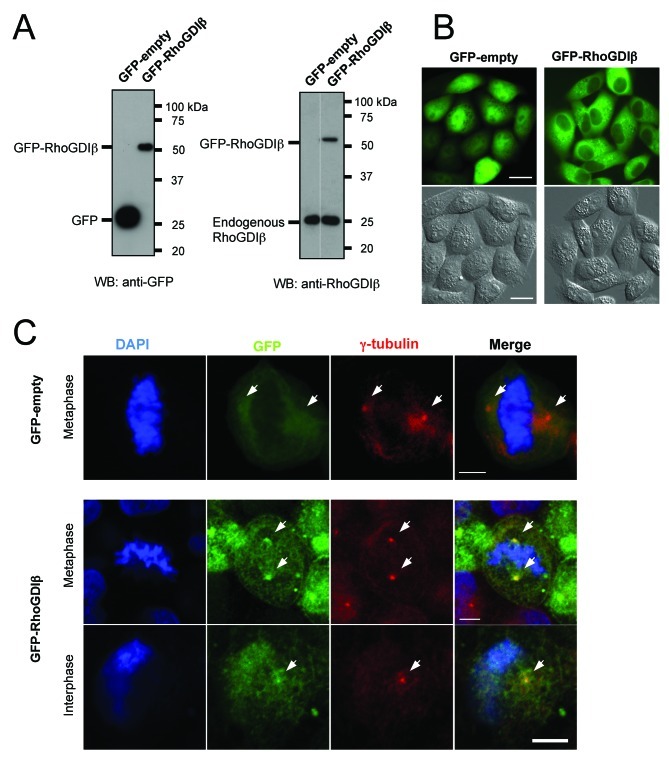
Subcellular localization of GFP-RhoGDIβ in HeLa cells. (A) Whole-cell lysates were prepared from HeLa cells expressing GFP-RhoGDIβ. The levels of the GFP-RhoGDIβ protein (left panel) and endogenous RhoGDIβ protein (right panel) were determined by immunoblotting analysis using anti-GFP and anti-RhoGDIβ (556498) antibodies. (B) GFP fluorescence images (upper panels) and DIC images (lower panels) of HeLa cells expressing GFP-RhoGDIβ. Scale bars, 20 *μ*m. (C) Cells were fixed with 4% paraformaldehyde, permeabilized with 0.5% Triton X-100, fixed again with 4% paraformaldehyde, then stained simultaneously with anti-GFP (green) and anti-γ-tubulin (red) antibodies and with DAPI (blue). Images were acquired on an LSM710 confocal microscope. Arrows indicate centrosomes. Scale bars, 5 *μ*m. Representative images are shown from at least three independent experiments.

We used GFP-RhoGDIβ to confirm the localization of RhoGDIβ to centrosomes. pAcGFP-RhoGDIβ was transfected into HeLa cells and the cells stably expressing the introduced genes were selected (Fig. 3A). Expression of GFP-RhoGDIβ did not affect the levels of endogenous RhoGDIβ protein (Fig. 3A, right panel). In living cells, localization of GFP-RhoGDIβ was distinct from localization of GFP only. Specifically, GFP-empty localized all over the cell, although it was brighter in the nuclei than in cell cytoplasm, while GFP-RhoGDIβ localized predominantly to the cytoplasm (Fig. 3B). It was difficult to observe centrosomal localization of GFP-RhoGDIβ in living cells because of its granular localization throughout the cytoplasm. To confirm the centrosomal localization of GFP-RhoGDIβ, cells were fixed and stained with both anti-GFP and anti-γ-tubulin antibodies. GFP-RhoGDIβ colocalized with γ-tubulin in both metaphase and interphase cells, while GFP-empty did not (Fig. 3C).

### Time-lapse observation of mitosis in HeLa cells stably expressing GFP-RhoGDIβ

**Figure 4.**
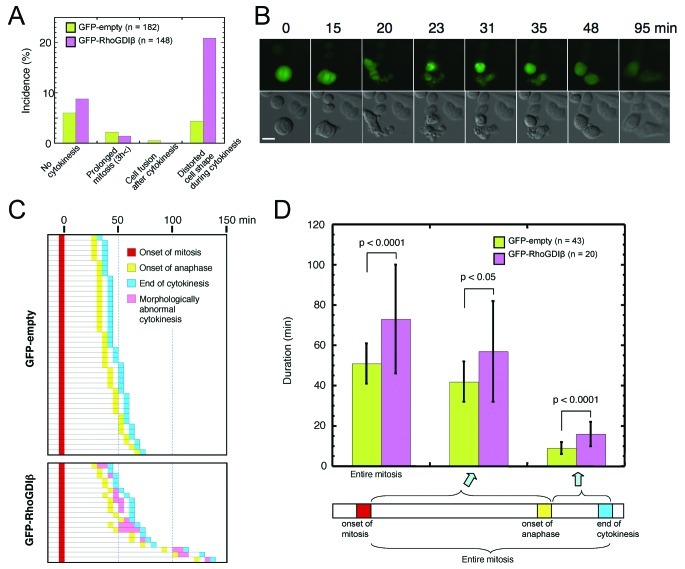
Time-lapse analysis of mitosis of HeLa cells expressing GFP-RhoGDIβ. Cells were cultured in 35-mm glass-bottomed dishes. GFP fluorescence images and DIC images were acquired on an Axiovert 200 M inverted microscope every 5 min for 30 h. (A) Incidence of mitotic abnormalities. (B) Representative images of severely distorted cell shape during cytokinesis of GFP-RhoGDIβ-expressing cell. Bars, 20 *μ*m. (C) Among the cells observed (A), the mitotic cells, in which the mitotic processes were clearly distinguished by the criteria as described in Materials and methods and the entire mitotic duration was less than 3 h, were analyzed. Horizontal columns indicate the mitotic progression of individual cells. n=43, GFP-empty; n=20, GFP-RhoGDIβ. (D) The mean values of mitotic duration of cells (C) were compared. Bars indicate SD. ^*^p<0.005 by the Mann-Whitney U-test.

Our observations suggested that RhoGDIβ had a role related to centrosome function. To investigate this, we used time-lapse microscopy to observe the mitotic progression in HeLa cells stably expressing GFP-RhoGDIβ. The incidence of morphologically aberrant cytokinesis, in which the cell shape was distorted, increased about 4-fold in GFP-RhoGDIβ expressing cells (Fig. 4A). Representative images of aberrant cytokinesis of GFP-RhoGDIβ-expressing cell are shown (Fig. 4B). To examine the defects in cytokinesis in detail we analyzed the time-lapse images of the cells, in which the onset of mitosis, the onset of anaphase, and the end of cytokinesis could be clearly distinguished (Fig. 4C). In these cells, morphologically aberrant cytokinesis was observed in 70% (14/20) of cells expressing GFP-RhoGDIβ, but was not observed in cells expressing GFP-empty. Duration from the onset of anaphase and from the onset of anaphase to the end of cytokinesis was significantly increased in cells expressing GFP-RhoGDIβ compared with those of cells expressing GFP-empty (Fig. 4D). These observations indicated that GFP-RhoGDIβ affected the mitotic processes including anaphase and cytokinesis in HeLa cells, suggesting that RhoGDIβ plays a role in these mitotic processes.

### Effect of knockdown of RhoGDIβ by siRNA

**Figure 5.**
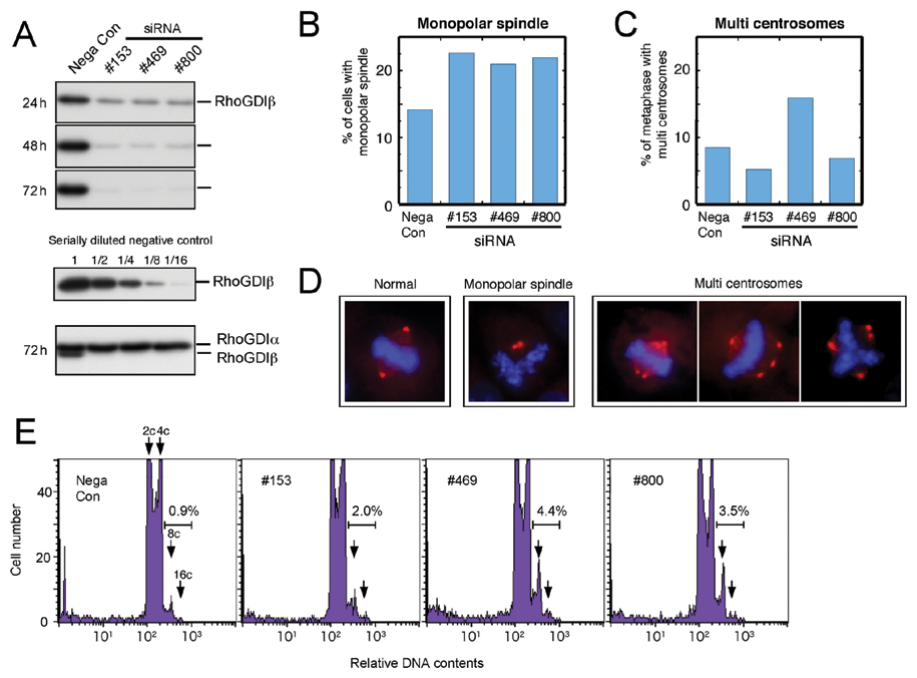
Effects of siRNA knockdown of RhoGDIβ in HeLa cells. (A) Stealth RNAi duplexes were transfected into HeLa cells and the protein levels of RhoGDIβ and RhoGDIα were determined by immunoblot analysis using anti-RhoGDIβ (556498) or anti-RhoGDIα/RhoGDIβ (66586E) antibodies at the indicated time after transfection. To estimate the degree of knockdown serially diluted samples of negative control were immunoblotted. Similar results were obtained from three independent experiments. (B and C) Cells were fixed with methanol 72 h after transfection of Stealth RNAi duplexes and stained with anti-γ-tubulin antibody (red) and DAPI (blue). The number of abnormal metaphases, such as (B) monopolar spindle and (C) multiple centrosomes, was counted under a fluorescence microscope. The total numbers of metaphases observed were 176, 229, 276 and 233 for the negative control, nos. 153, 469 and 800, respectively. Similar results were obtained from two independent experiments. (D) Representative images of aberrant position and number of centrosomes observed in RhoGDIβ knockdown cells. (E) DNA histograms were obtained by flow cytometry 72 h after transfection of Stealth RNAi duplexes. Similar results were obtained from three independent experiments.

We examined the effect of RhoGDIβ knockdown in HeLa cells using three different siRNAs. All siRNAs decreased the expression of RhoGDIβ by about 90% and did not decrease the expression of RhoGDIα 72 h after transfection (Fig. 5A). Knockdown of RhoGDIβ by all three siRNAs increased the incidence of monopolar spindle mitosis (Fig. 5B). In contrast with the appearance of monopolar mitotic cells, a slightly higher frequency of multiple centrosomes was observed only in cells that had RhoGDIβ knocked down by no. 469 siRNA (Fig. 5C). Representative images of monopolar spindle mitosis and multiple centrosomes mitosis are shown (Fig. 5D). Therefore, we concluded that RhoGDIβ knockdown induced inhibition of centrosome separation rather than multipolar mitosis. The increase of polyploid (8, 16c or more) cells after RhoGDIβ knockdown as shown in Fig. 5E were thought to mainly result from monopolar mitosis. Overall, our data show that both upregulation and downregulation of RhoGDIβ constitute key events leading to perturbed mitotic processes in cancer cells.

## Discussion

RhoGDIβ is abundantly expressed in hematopoietic cells (11), but is also expressed in various non-hematopoietic cells (13–17). Using immunoblotting, we confirmed that RhoGDIβ protein is expressed in many human epithelial cell lines as well as in many mouse organs, suggesting a general role for RhoGDIβ that is not specific to hematopoietic cells. We showed that RhoGDIβ, but not RhoGDIα, localized to centrosomes in human colon cancer cells, human keratinocytes and HeLa cells by immunofluorescence staining. Furthermore, we showed that exogenously introduced GFP-RhoGDIβ also localized to centrosomes in HeLa cells. Previously, RhoGDIβ was identified in the purified centrosome fraction from a human lymphoblastic cell line by the proteomic analysis, however, it was not confirmed as a genuine centrosomal protein (32). Since RhoGDIβ functions as a chaperone (8), its association with the centrosome would be expected to be transient and less stable than those of the scaffold proteins of centrosomes. Furthermore, RhoGDIβ is abundant in the cytosol. These properties of RhoGDIβ make it difficult to clarify its localization to the centrosome. In the present study, we confirmed that RhoGDIβ localized to centrosomes and suggested that RhoGDIβ had a role related to centrosome function. Actually, we showed that the expression of GFP-RhoGDIβ significantly prolonged anaphase and cytokinesis and increased the morphologically aberrant cytokinesis in HeLa cells. Furthermore, RhoGDIβ knockdown caused defects in centrosome separation. Supporting a previous proteomics study (32), collectively, our present data regarding localization and functional analyses strongly suggest that RhoGDIβ functions in centrosomes during mitosis.

Rho family proteins are important regulators of both cytokinesis and centrosome positioning (33). RhoA is the central regulator of cytokinesis in animal cells (34). Cdc42 and MgcRacGAP are reported to contribute to the correct formation of mitotic spindles during metaphase in HeLa cells (35). Rac and Tiam1 are localized to the centrosomal regions during early mitosis, and bipolar spindle formation is regulated by Tiam1-Rac signaling in MDCK II cells (36). Therefore, our results collectively suggest that RhoGDIβ, that is a regulator for Rho proteins, plays a role in the regulation of cytokinesis and the formation of bipolar spindles. To our knowledge this is the first report suggesting that RhoGDIβ participates in the regulation of these mitotic processes.

In normal cells, the mitotic checkpoint prevents the transition to anaphase when the monopolar spindle is formed; however, in cancer cells, this checkpoint is compromised and some cells become polyploid through aberrant mitosis (37). Therefore, an increase of polyploid cells by knockdown of RhoGDIβ could be due to the increased incidence of monopolar spindle. The monopolar spindles can result from defects in many different molecules, such as specific motor proteins, centrosome proteins, and mitotic kinases (38). Which of these molecules are associated with the observed phenotype by RhoGDIβ knockdown is unknown, but RhoGDIβ should be involved in the formation of normal bipolar spindles when required.

Correct centriole and centrosome positioning is important for many biological processes (39) and centrosomes play important roles in maintaining the polarity axis during asymmetrical cell division, and disruption of polarity is implicated in cancer development and progression (40–42). Cell motility, invasion, and anoikis in cancer progression are regulated by RhoGDIβ in cancer progression, irrespective of the direction of correlation with RhoGDIβ expression (14,15,19,21,23,25,27) and are closely associated with cell polarity (43,44). There is crosstalk between Rho family proteins and polarity proteins (44). The roles of RhoGDIβ in cancer progression may be related, at least in part, to its role in the regulation of cell polarity. RhoGDIs are conserved among eukaryotes and are suggested to have a universal role in the regulation of cell polarity in a wide range of eukaryotes (45–51). Many unicellular eukaryotes and lower metazoa have a single RhoGDI (52), while vertebrates, except for bony fish, have three kinds of RhoGDIs. Our results suggest that among RhoGDIs at least RhoGDIβ plays a role in the regulation of cell polarity in mammalian cells.
